# Optimization of treatment strategies based on preoperative imaging features and local recurrence areas for locally advanced lower rectal cancer after lateral pelvic lymph node dissection

**DOI:** 10.3389/fonc.2023.1272808

**Published:** 2024-02-05

**Authors:** Zhao Xu, Mandula Bao, Qiang Cai, Qian Wang, Wei Xing, Qian Liu

**Affiliations:** ^1^Department of General Surgery, Hebei Province Hospital of Chinese Medicine, Affiliated Hospital of Hebei University of Chinese Medicine, Shijiazhuang, China; ^2^Department of Colorectal Surgery, National Cancer Center/National Clinical Research Center for Cancer/Cancer Hospital, Chinese Academy of Medical Sciences and Peking Union Medical College, Beijing, China; ^3^Department of Gastric and Colorectal Surgery, General Surgery Center, The First Hospital of Jilin University, Changchun, China; ^4^Department of Oncology, Shanghai Medical College, Fudan University, Shanghai, China

**Keywords:** lateral pelvic lymph node dissection, rectal cancer, local recurrence, neoadjuvant chemoradiotherapy, circumferential resection margin

## Abstract

**Purpose:**

Local recurrence (LR) is the main cause of treatment failure in locally advanced lower rectal cancer (LALRC). This study evaluated the preoperative risk factors for LR in patients with LALRC to improve the therapeutic strategies.

**Patients and Methods:**

LALRC patients who underwent total mesorectal excision (TME) with lateral pelvic lymph node (LPN) dissection (LPND) from January 2012 to December 2019 were reviewed. The log-rank test was used to assess local recurrence-free survival (LRFS), and multivariate Cox regression was used to identify the prognostic risk factors for LRFS. Follow-up imaging data were used to classify LR according to the location.

**Results:**

Overall, 376 patients were enrolled, and 8.8% (n=33) of these patients developed LR after surgery. Multivariate analysis identified positive clinical circumferential resection margin (cCRM) as an independent prognostic factor for LRFS (HR: 4.94; 95% CI, 1.75-13.94; *P*=0.003). The most common sites for LR were the pelvic plexus and internal iliac area (PIA) (54.5%), followed by the central pelvic area (CPA) (39.4%) and obturator area (OA) (6.1%). Following a subgroup analysis, LR in the OA was not associated with positive cCRM. Patients treated with upfront surgery (n=35, 14.1%) had a lower cCRM positive rate when compared with patients treated with neoadjuvant chemoradiotherapy (nCRT) (n=12, 23.5%). However, the LR rate in the nCRT group was still lower (n=28, 36.4%) than that in the upfront surgery group (n=35, 14.%). Among patients with positive cCRM, the LR rate in patients with nCRT remained low (n=3, 10.7%).

**Conclusion:**

Positive cCRM is an independent risk factor for LR after TME plus LPND in LALRC patients. LPND is effective and adequate for local control within the OA regardless of cCRM status. However, for LALRC patients with positive cCRM, nCRT should be considered before LPND to further reduce LR in the PIA and CPA.

## Introduction

Since the introduction of total mesorectal excision (TME) in 1982, the local recurrence (LR) rate of rectal cancer was significant decreased (1, 2). However, the local control in patients treated with TME alone for locally advanced lower rectal cancer (LALRC) is still not satisfactory (3). The use of neoadjuvant chemoradiotherapy (nCRT) and lateral pelvic lymph node (LPN) dissection (LPND) to reduce LR in these patients is still controversial. The Japanese guidelines recommend the adoption of TME with prophylactic LPN dissection (LPND) to treat LALRC (T3/T4) (4). The Japanese clinical oncology group (JCOG) 0212 large-scale clinical trial demonstrated that TME with LPND reduced the LR in patients diagnosed with lateral tumors when compared with TME alone (7.4% versus 12.6%, *P*=0.024) *(* 5). Conversely, for LALRC, the European guidelines recommend the use of nCRT instead of LPND to eradicate lateral disease and reduce the risk of LR (6, 7). However, studies have shown that the LR rate ranged between 7.2% and 13.7% after nCRT followed by TME, and the proportion of lateral pelvis recurrence is as high as 64.6%-82.7% (8). Furthermore, a multicenter collaborative study on LPNs showed that nCRT alone without LPND cannot completely eradicate metastatic LPNs. An additional LPND could significantly reduce recurrence within the lateral compartment (9). Therefore, LPND has a positive significance in improving LR in patients with LALRC.

However, some studies reported that LR rate remained between 5% to 10% after TME with LPND for LALRC patients with clear margins (5, 10). In recent years, the value and significance of comprehensive treatment strategies in LALRC have gradually emerged, and surgeons are now evaluating the use of nCRT before LPND to further reduce the risk of LR (11–13). However, immunosuppression and tissue edema caused by nCRT also increase the management difficulty and risk of complications following LPND. This highlights the need to identify factors leading to LR after LPND in patients with LALRC to optimize the treatment for these patients.

Therefore we conducted a multicenter retrospective study to identify the impact of preoperative clinical characteristics and radiologic features on the risk of developing LR after LPND in patients with LALRC. Meanwhile, the areas of LR and different preoperative treatment methods were analyzed in order to tailor appropriate comprehensive management approach according to different recurrence risk groups to improve the LR of patients with LALRC.

## Methods

### Study population

LALRC patients who underwent TME with LPND from January 2011 to December 2019 at three institutions of the Chinese Lateral Node Collaborative Group were identified. The clinical and radiographic characteristics were retrospectively extracted from the institutional databases and tumor registries.

The patients were included in the study if they underwent standard LPND according to the Japanese Society for Cancer of the Colon and Rectum (JSCCR) guidelines for a clinical advanced rectal cancer (cT3-T4/cN+) and pathologically confirmed adenocarcinoma with the lower margin of the tumor located below the peritoneal reflection. All enrolled patients were followed for at least 36 months. Patients who underwent a palliative resection were excluded. In addition, patients with a history of other malignancies, incomplete follow-up data, and/or distant metastases were also excluded.

### Ethical consideration

The study was conducted in accordance with the ethical standards of the World Medical Association Declaration of Helsinki and the STROBE Guidelines. The institutional ethics review boards of the three participating hospitals approved the study. The trial was registered (NCT04850027) in ClinicalTrials.gov. Written informed consent was obtained from all patients enrolled in the study.

### Preoperative diagnosis

All patients underwent preoperative examination, including colonoscopy, serum tumor marker analysis, computed tomography (CT), and pelvic magnetic resonance imaging (MRI). The images were evaluated by two radiologists who specialized in colorectal cancer, and the TNM stage, clinical circumferential resection margin (cCRM), extramural venous invasion (EMVI), and LPN status were recorded. Positive cCRM was defined as a distance below or equal to 1 mm between the tumor and mesenteric fascia or levator muscle (14). The EMVI status was assessed according to a 5-scale EMVI scoring system (15), whereby a score between 0 to 2 was defined as negative, and a score of 3 and 4 was defined as positive. The American Joint Committee on Cancer (AJCC) staging system (8th edition) was used to assess the TNM staging (16).

### Treatment strategies

The indication of LPND was determined for the patients with cT4, cN2, or clinical suspected LPN metastasis. Clinically suspicious LPN metastasis was defined as a node with the shortest axis diameter above or equal to 5 mm with inhomogeneous or intense enhancement and an irregular shape with rough edges based on MRI. The treatment strategies for the patients were determined based on the patient’s preferences and the recommendations of a multidisciplinary team that incorporated radiologists, medical oncologists, and surgical oncologists. nCRT or neoadjuvant chemotherapy (NAC) was recommended for patients with a high risk of distant metastasis or LR, such as T4b stage and multiple lymph node metastases. The indications for the use of nCRT and NAC were similar. Treatment strategies for LPN metastases were updated during the study period. Between 2011 and 2017, patients with clinically suspected LPN metastasis were mainly treated using upfront surgery without preoperative treatment. After 2018, preoperative chemotherapy or chemoradiation was performed before LPND for patients with a short LPN diameter above 8 mm.

The nCRT regimen consisted of a long radiotherapy treatment course using a prescription of 50 Gy in 25 fractions and capecitabine at a dose of 825 mg/m^2^ administered twice daily on all days of radiotherapy. The NAC regimen consisted of 4 to 6 cycles of either FOLFOX or XEOLX. Surgical resections were performed 4 to 6 weeks after NAC and 6 to 8 weeks after nCRT.

### LPND procedure

All chief surgeons involved in the study had completed at least 500 cases of laparoscopic colorectal surgery and mastered the mature LPND technique. Unilateral LPND was usually performed according to the location of enlarged LPN or the main invasion direction of the primary tumor, while bilateral LPND was only performed in bilateral LPN enlargement. The LPND was performed in accordance with the JSCCR guidelines for all patients (13, 17). The extent of the dissection included 4 areas: the internal iliac lymph node, obturator lymph node, external iliac lymph node, and common iliac lymph node (4).

### Adjuvant therapy

Patients with stage III and high-risk stage II diseases (CRM ≤ 1 mm, pT4, tumor perforation, lymphatic invasion, perineural invasion) received adjuvant chemotherapy within 4 to 6 weeks after surgery. All patients treated with nCRT, irrespective of their pathological stage, received 6 months of perioperative chemotherapy.

### Follow-up procedure

Patients were followed-up every 3 months for the first 3 years and every 6 months after 3 years. The examinations performed during each follow-up included a physical examination, assessment of tumor markers (CEA and CA19-9), and a CT of the chest, abdomen, and pelvis. In addition, a total endoscopy was performed annually. The endpoints of this study were 3-year LR-free survival (LRFS) and 3-year LR rate.

### Classification of LR area

**Figure 1:**
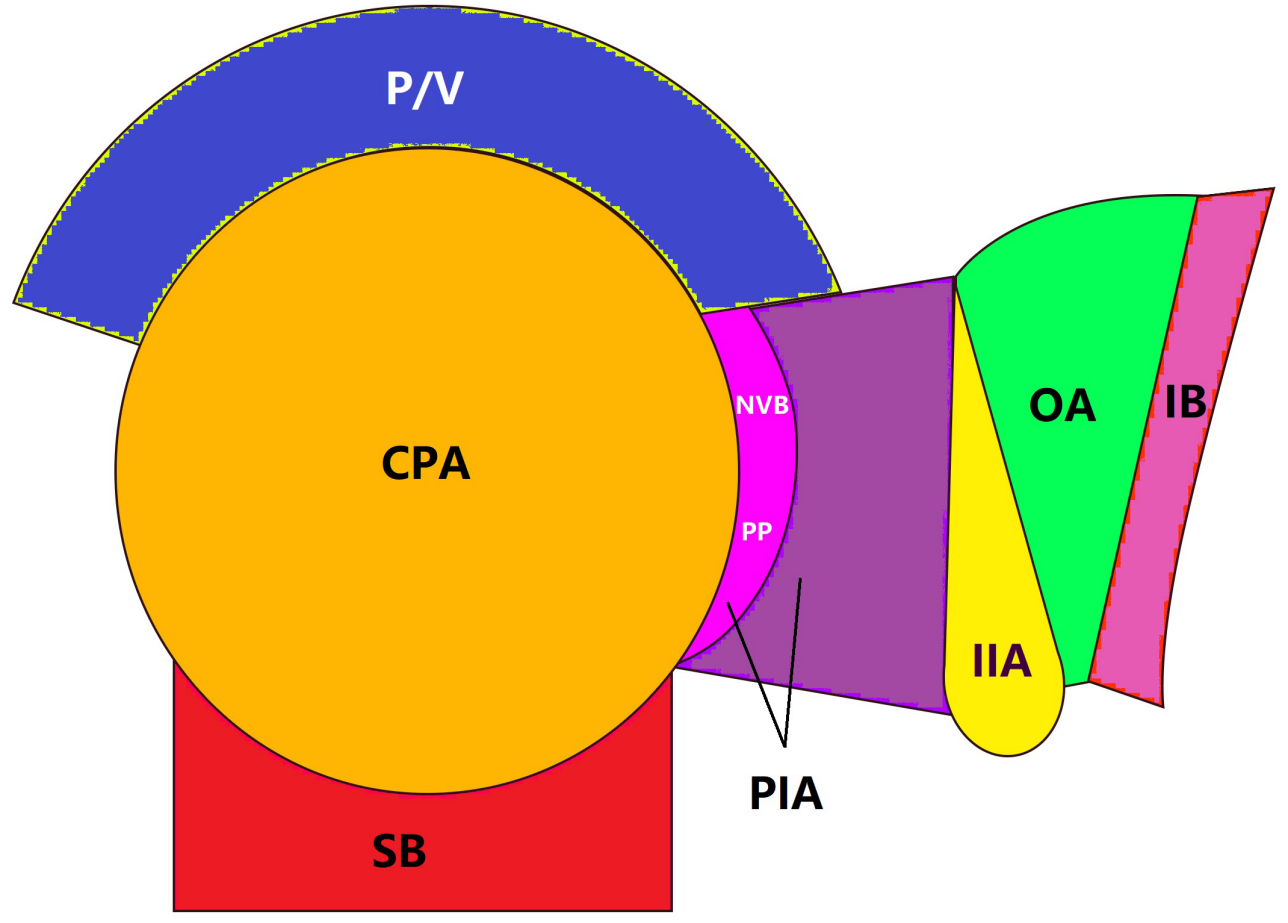
Local recurrence areas. Local recurrence was classified into 3 areas: CAP, central pelvic areal; P/V, prostate or vagina; SB, sacrum bone; PIA, pelvic plexus and internal iliac area; NVB, neurovascular bundle; PP, pelvic plexus; IIA, internal iliac artery; OA, obturator area; IB, ischial bone.

**Figure 2:**
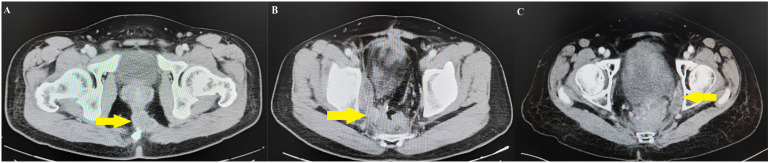
Follow-up image data of local recurrence areas. **(A)** Central pelvis area case identified the local recurrence on presacral fascia. **(B)** Pelvic plexus and internal iliac area case identified the local recurrence between the right pelvic plexus and the internal iliac artery. **(C)** Obturator area case identified the local recurrence between internal iliac artery and pelvic side wall.

All patients were followed for more than 3 years, so only LR within 3 years were counted in this study. The LR sites were classified into central pelvis area (CPA), pelvic plexus and internal iliac area (PIA), or obturator area (OA) based on follow-up image data as described by Shiraishi et al. (18) (**Figure 1**). LR within the anastomosis region, presacral fascia, or perirectal soft tissue away from the pelvic plexus and neurovascular bundle was classified as CPA (**Figure 2A**), LR within the soft tissue area around the pelvic plexus or neurovascular bundle or the region along the internal iliac artery and veins was classified as PIA (**Figure 2B**), and LR between the internal iliac artery and pelvic sidewall was classified as OA (**Figure 2C**).

### Statistical analysis

The continuous variables were expressed as mean ± standard deviation and compared using the paired T-test or Mann–Whitney’s U-test. The categorical variables are presented as percentages, and the Chi-square test or Fisher’s exact test was used to compare these variables. The 3-year cumulative LRFS was calculated by the Kaplan–Meier method, and univariate analysis was performed using the log-rank test. The statistically significant variables in the univariate analysis were subsequently tested by multivariate analysis using a Cox regression model. A *P*-value below 0.05 was considered statistically significant. The statistical package for social science (SPSS) software version 24.0 (IBM Inc., Armonk, NY, USA) was used for statistical analysis.

## Results

### Patient characteristics

A total of 376 patients were eligible for the study, of whom 33 had LR and 343 did not have LR. The patients’ demographic data and clinical characteristics are summarized in **Table 1**. The cT stage, cN stage, enlarged LPN, cCRM, EMVI, and adjuvant therapy differed significantly between the LR and non-LR groups. (*P*<0.05).

**Table 1:** The demographic data and clinical characteristics between LR and non-LR groups.

Characteristics	LR (n=33)	Non-LR (n=343)	*P*
Age at operation (y, mean ± SD)	54.1 ± 12.2	57.2 ± 11.3	0.143
Gender (%)			0.340
Male	18 (54.5)	216 (63.0)	
Female	15 (45.5)	127 (27.0)	
BMI (Kg/m^2^,mean ± SD)	23.3 ± 2.4	24.5 ± 5.6	0.228
ASA score (%)			1.000
I-II	32 (97.0)	333 (97.1)	
III	1 (3.0)	10 (2.9)	
Preoperative CEA level (ng/ml)			0.723
<5	22 (66.7)	218 (63.6)	
≥5	11 (33.3)	125 (36.4)	
Distance from AV (cm, mean ± SD)	4.3 ± 2.5	4.8 ± 2.4	0.333
Preoperative treatment			0.013
None	18 (54.5)	230 (67.1)	
Preoperative chemotherapy	10 (30.3)	41 (12.0)	
Preoperative Chemoradiotherapy	5 (15.2)	72 (20.9)	
cT stage (%)			0.022
T1-T2	0 (0)	44 (12.8)	
T3-T4	33 (100.0)	299 (87.2)	
cN stage (%)			0.012
N0	3 (9.1)	102 (29.7)	
N1-N2	30 (90.9)	241 (70.3)	
Enlarged LPN			0.042
Presence	19 (57.6)	135 (39.4)	
Absence	14 (42.4)	208 (60.6)	
EMVI			0.011
Positive	10 (30.3)	47 (13.7)	
Negative	23 (69.7)	296 (86.3)	
cCRM			<0.001
Positive	17 (51.5)	58 (16.9)	
Negative	16 (48.5)	285 (83.1)	
Adjuvant therapy			0.027
Presence	29 (87.9)	239 (69.7)	
Absence	4 (12.1)	104 (30.3)	

### Surgical outcomes and postoperative recovery

The surgical outcomes and postoperative recovery are shown in **Table 2**. The majority of the patients (n=267, 71.0%) had laparoscopic surgery as opposed to open surgery. The surgery involved either a low anterior resection (n=192, 51.1%), abdominoperineal resection (n=147, 39.1%), Hartmann procedure (n=12, 3.2%), or a total pelvic exenteration (n=25, 6.6%). There were no significant differences in the operation type, resection site, LPND procedure operation time, intraoperative blood loss, postoperative complications, and length of hospital stay between the LR and non-LR groups (*P*>0.05).

**Table 2:** The surgical outcomes and postoperative recovery between LR and non-LR groups.

Characteristics	LR (n=33)	Non-LR (n=343)	*P*
Operation type			0.303
Open	7 (21.2)	102 (29.7)	
Laparoscopic	26 (78.8)	241 (70.3)	
Surgical procedure			0.271
Low anterior resection	12 (36.4)	180 (52.5)	
Abdominoperineal resection	17 (51.4)	130 (37.9)	
Hartmann procedure	2 (6.1)	10 (2.9)	
Total pelvic exenteration	2 (6.1)	23 (6.7)	
LPND procedure			0.497
Unilateral dissection	19 (57.6)	218 (63.6)	
Bilateral dissection	14 (42.4)	125 (36.4)	
Operation time (min, mean ± SD)	303.3 ± 82.1	290.4 ± 112.1	0.519
Estimated blood loss (ml, mean ± SD)	214.6 ± 269.0	237.2 ± 379.8	0.739
Postoperative complications			0.695
Presence	9 (27.3)	83 (24.1)	
Absence	24 (72.7)	260 (75.9)	
Total hospital stay (day, mean ± SD)	12.3 ± 12.0	13.9 ± 12.6	0.695

### Pathological results

The pathological results between the LR and non-LR groups are compared in **Table 3**. The proportion of patients in the LR group with T3-T4 stage (90.9% versus 68.8%, *P*=0.008), N1-N2 stage (78.8% versus 45.2%, *P*<0.001), poor differentiation (42.4% versus 25.7%, *P*=0.039), and perineural invasion (60.6% versus 36.7%, *P*=0.007) was significantly higher than that in the non-LR group.

**Table 3:** Pathological results between LR and non-LR groups.

Characteristics	LR (n=33)	Non-LR (n=343)	*P*
(y)pT stage			0.008
T0-T2	3 (9.1)	107 (31.2)	
T3-T4	30 (90.9)	236 (68.8)	
(y)pN stage			<0.001
N0	7 (21.2)	188 (54.8)	
N1-N2	26 (78.8)	155 (45.2)	
Histologic grade			0.039
Moderate	19 (57.6)	255 (74.3)	
Poor/Mucinous/signet	14 (42.4)	88 (25.7)	
Pathological LPNM			0.233
Presence	10 (30.3)	73 (21.3)	
Absence	23 (69.7)	270 (78.7)	
CRM status			0.188
Positive	4 (12.1)	17 (5.0)	
Negative	29 (87.9)	326 (95.0)	
Perineural invasion			0.007
Presence	20 (60.6)	126 (36.7)	
Absence	13 (39.4)	217 (63.3)	
Lymphatic invasion			0.057
Presence	15 (45.5)	101 (29.4)	
Absence	18 (54.5)	242 (70.6)	
LPNs removed (n, mean ± SD)	7.3 ± 4.9	9.0 ± 6.2	0.120
Mesorectal LN removed (n, mean ± SD)	16.8 ± 6.7	17.4 ± 8.2	0.422

### Preoperative risk factors for LR and LRFS

**Figure 3:**
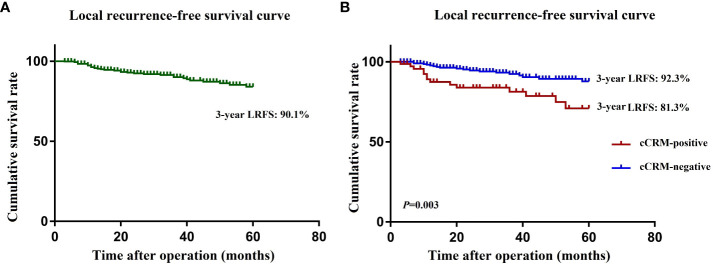
**(A)** Kaplan-Meier curves for local recurrence rate in all patients. **(B)** Kaplan-Meier curves for local recurrence rate in cCRM-positive and cCRM-negative patients. LRFS, local recurrence free survival; cCRM, clinical circumferential resection margin.

The median follow-up period for the entire group was 57 months. The incidence of 3-year LR was 8.8% (33/376), and the estimated 3-year LRFS rate was 90.1% (**Figure 3A**). The univariate and multivariate analyses of the preoperative risk factors influencing LRFS are presented in **Table 4**. The 3-year LRFS in positive cCRM patients was significantly worse than patients with negative cCRM (92.3% versus 81.3%, *P*=0.003) (**Figure 3B**). Univariate analysis demonstrated that enlarged LPN, histology, EMVI, cCRM, cT stage, and cN stage were the preoperative predictors for LRFS (*P*<0.05). Multivariate analysis revealed positive cCRM (HR: 4.94; 95% CI, 1.75-13.94; *P*=0.003) as an independent prognostic factor for LRFS.

**Table 4:** The univariate and multivariate analyses of the preoperative risk factors influencing LRFS.

Variables	Local recurrence-free survival			
	Univariate analysis		Multivariate analysis	
	HR (95%CI)	*P*	HR (95%CI)	*P*
Gender: male/female	0.67 (0.34-1.34)	0.259		
Age at operation (≥65/<65years)	0.68 (0.28-1.64)	0.384		
CEA level (>5/≤5 ng/L)	1.13 (0.55-2.33)	0.748		
Distance from anal verge (>5/≤5 cm)	0.58 (0.26-1.29)	0.184		
Preoperative treatment (yes/no)	1.09 (0.69-1.72)	0.718		
None				
Preoperative chemotherapy	1.90 (0.88-4.11)	0.105		
Preoperative Chemoradiotherapy	1.46 (0.35-2.41)	0.464		
Enlarged LPN	2.80 (1.39-5.64)	0.004	1.83 (0.69-4.86)	0.226
Histology ( Poor, Mucinous or signet/moderate)	2.59 (1.16-5.80)	0.021	1.88 (0.79-4.47)	0.152
EMVI (Positive/negative)	2.38 (1.15-4.92)	0.019	1.67 (0.49-5.65)	0.409
cCRM (Positive/negative)	2.81 (1.41-5.62)	0.003	4.94 (1.75-13.94)	0.003
cT stage	1.73 (1.05-2.99)	0.048	0.86 (0.46-1.60)	0.630
T1-T2	Reference		Reference	
T3	2.03 (0.77-5.36)	0.153	1.24 (0.49-3.35)	0.626
T4	2.85 (1.28-8.70)	0.010	1.86 (0.91-7.32)	0.102
cN stage				
N0	Reference		Reference	
N1	1.77 (0.77-4.11)	0.181	0.89 (0.27-2.29)	0.840
N2	2.71 (1.19-6.17)	0.017	1.22 (0.43-3.41)	0.712

### LRFS and LR according to cCRM status, tumor site resection, and preoperative treatment

The most common LR site was PIA (n=18, 54.5%), followed by CPA (n=13, 39.4%) and OA (n=2, 6.1%). **Table 5** summarizes the findings of the subgroup analysis evaluating the relationship between LR site and cCRM. The proportion of positive cCRM in patients with LR in the CPA (8.3% *vs* 2.3%, *P*=0.022) and PIA (13.9% *vs* 2.6%, *P*<0.001) were significantly higher, suggesting that LR in these two areas were associated with positive cCRM. LR in the OA was not associated with positive cCRM. However, the number of LR events in the OA was too small.

**Table 5:** Relationship between LR area and cCRM.

Local recurrence area	cCRM		*P*
	Positive (n=72)	Negative (n=304)	
Central pelvis area	6 (8.3)	7 (2.3)	0.022
Pelvic plexus and internal iliac area	10 (13.9)	8 (2.6)	<0.001
Obturator area	1 (1.4)	1 (0.3)	0.347

**Table 6** shows the LR and cCRM status according to the preoperative treatment. LR was observed in 18 (7.3%) of the 248 patients treated with upfront surgery, 10 (19.6%) of the 51 patients treated with NAC, and 5 (6.5%) of the 77 patients treated with nCRT. Patients who underwent nCRT had a lower LR rate. However, it is important to note that the cCRM positive rate in patients who underwent nCRT (n=28, 36.4%) was higher than that of patients treated with upfront surgery (n=35, 14.1%) and NAC (n=12, 23.5%). In addition, among patients with positive cCRM, the LR rate in patients who underwent nCRT remained low (n=3, 10.7%).

**Table 6:** LR and cCRM status according to preoperative treatment.

Local recurrence area	Preoperative treatment		
	None (n=248)	NAC (n=51)	NCRT (n=77)
Local recurrence	18 (7.3)	10 (19.6)	5 (6.5)
cCRM positive	35 (14.1)	12 (23.5)	28 (36.4)
Local recurrence in cCRM positive	10/35 (28.6)	4/12 (33.3)	3/28 (10.7)

## Discussion

The JCOG0212 trial demonstrated the benefits of LPND in LALRC patients in reducing LR in the lateral pelvic compartment following TME (5). However, even in patients with negative surgical margins, the LR rate remains high at 5% to 10% after TME with LPND for LALRC patients (5, 10). Therefore, this study aimed to explore the preoperative risk factors associated with LR after TME with LPND. In addition, the LR sites were also classified and evaluated in detail to selected appropriate comprehensive management approach in specific patients.

Primarily, our study demonstrated that the 3-year LRFS in positive cCRM patients was significantly worse than that in patients with negative cCRM. (92.3% versus 81.3%, *P*=0.003). A positive cCRM was identified as an independent prognostic factor (HR: 4.94; 95% CI, 1.75-13.94; *P*=0.003) for LRFS. In addition, the highest LR rate occurred in the PIA (n=18, 54.5%), while LR in the OA (n=2, 6.1%) was the least common. Patients with positive cCRM had a high LR rate in the PIA and CPA but not in the OA. LPND followed by nCRT might effectively reduce the risk of LR in the PIA and CPA, especially in cases with positive cCRM.

Lateral lymphatic drainage is one of the most common metastatic pathways for tumors located below the peritoneal reflection. Our previous study demonstrated that patients with pathologically confirmed LPN metastasis could achieve satisfactory local control through LPND (19). This study identified LPN enlargement in 154 (40.9%) preoperative MRIs, which were subsequently confirmed by pathology in 83 (22.1%) patients. Univariate analysis showed that LPN enlargement was associated with worse LRFS (*P*=0.004). However, multiple lymph node metastases, poor differentiation, positive cCRM, and other adverse pathological factors can increase the risk of LR after surgery. Enlarged LPN was not an independent prognostic factor for LRFS after the above confounders were eliminated by multivariate analysis, suggesting that LPND can achieve satisfactory local control for patients with enlarged LPN.

Studies have shown that the accuracy of the preoperative cCMR assessment on MRI is comparable to that of the pathological gold standard (14, 18, 20). The present study revealed that positive cCRM was significantly associated with LR after LPND in patients with LALRC. Furthermore, the LR rate in the lateral pelvic area, except for LR in the OA, was high after LPND, especially in patients with positive cCRM. These findings suggest that the LPND procedure within the OA is easier to perform, minimizing the risk of residual microscopic disease after surgery. The lower local control rates in the PIA after TME with LPND in patients with positive cCRM could be attributed to cancer cells remaining in the pelvic plexus area when the tumor penetrates the proper fascia of the rectum via the lateral pathway. The LPND procedure that preserves the pelvic plexus may increase the possibility of residual tumor tissue omission, potentially increasing the risk of recurrence in this area. Therefore, multidisciplinary strategies should be considered for patients with positive cCRM to improve local control in the lateral pelvic area (PIA), except for the OA.

In this study, all enrolled patients were followed for at least 36 months, and the median follow-up period for the entire group was 57 months. Because the median follow-up was less than 5 years, therefore, the endpoints of this study were 3-year LRFS and 3-year LR rate. The literature has reported that the 3-year LR rate of patients with LALRC after R0 resection is 5%-14% (21–23). This study also included patients with LALRC (cT4, cN2, or clinical suspected LPN metastasis), and 3-year LR rate after radical surgery was 8.8%, which was basically consistent with previous literature reports. Neoadjuvant therapy has positive prognostic value and significance in patients with LALRC (24, 25). From 2009 to 2010, Poulsen et al. treated 479 (29%) of 1633 patients with LALRC in Denmark, and only 68 patients (4.2%) developed local recurrence within 3 years, reflecting the satisfactory local control effect of neoadjuvant therapy (26). In present study, we also analyzed the relationship between different preoperative treatment models and LR and found that patients with nCRT (6.5%) had a significantly lower LR rate than patients who underwent NAC (19.6%) and upfront surgery (7.3%), even if the proportion of positive cCRM was higher in patients with nCRT (36.4%). In addition, nCRT still has an advantage over NAC in terms of local control in patients with positive cCRM (10.7% versus 33.3%). Similarly, a previous study reported that NAC could not control the LR in patients with a high risk of recurrence (27). In addition, Shiraishi et al. also revealed that LALRC patients with positive cCRM required nCRT instead of NAC to decrease LR (15). Therefore, nCRT should be considered as preoperative treatment in LALRC patients with positive cCRM.

## Limitations

Our study has several limitations that have to be acknowledged. First of all, due to the limited number of participants, the study is prone to selection bias caused by variations in the population characteristics, surgical quality, and treatment strategies. The retrospective multicenter nature of the study may also limit the generalizability of the research findings. However, all three institutions involved in the study were tertiary hospitals from the Chinese Lateral Lymph Node Collaboration group. Therefore the treatment concept and technology can fully reflect the current diagnosis and treatment level of LPN metastasis in China. Moreover, the proportion of patients undergoing open surgery was higher in earlier years, and more recently the proportion undergoing laparoscopic surgery was higher. Changes in medical technology and treatment strategies can lead to different outcomes. In addition, while the indications for NAC and nCRT are similar, the surgeons’ preference for treatment may have influenced the research findings. Furthermore, since the study was performed over 8 years, the continuous development and updating of laparoscopic equipment and technology may have influenced the results of this study. Finally, the median follow-up time of the whole study was only 37 months, so we only calculate 3-year LRFS. A longer follow-up period is required to identify the long term impact of these treatments on LR and survival.

## Conclusions

The present study showed that positive cCRM is an independent risk factor for LR after TME with LPND in patients with LALRC. LPND is effective and adequate for local control in OA regardless cCRM status. However, for LALRC patients with positive cCRM, nCRT should be considered before LPND to further reduce LR in the PIA and CPA regions.

## Data availability statement

The raw data supporting the conclusions of this article will be made available by the authors, without undue reservation.

## Ethics statement

The studies involving humans were approved by All enrolled patients sign written informed consent to participate in the study. The study was conducted per STARD reporting guidelines. All the procedures followed the ethical standards of the World Medical Association Declaration of Helsinki. The study was approved by the institutional review boards of the three hospitals, and the study design was registered (NCT04850027) at ClinicalTrials. gov. The studies were conducted in accordance with the local legislation and institutional requirements. The participants provided their written informed consent to participate in this study.

## Author contributions

ZX: Conceptualization, Data curation, Investigation, Methodology, Writing – original draft. MB: Writing – original draft, Writing – review & editing, Conceptualization. QCC: Conceptualization, Data curation, Formal analysis, Methodology, Writing – original draft. QC: Supervision, Validation, Writing – review & editing. QW: Formal analysis, Investigation, Project administration, Software, Writing – review & editing. WX: Conceptualization, Funding acquisition, Supervision, Visualization, Writing – review & editing. QL: Formal analysis, Funding acquisition, Methodology, Supervision, Writing – review & editing.

## References

[1] Ortholan C Francois E Thomas O Benchimol D Baulieux J Bosset JF. Role of radiotherapy with surgery for T3 and resectable T4 rectal cancer: evidence from randomized trials. Dis Colon Rectum (2006) 49(3):302–10. doi: 10.1007/s10350-005-0263-x 16456638

[2] Bonjer HJ Deijen CL Abis GA Cuesta MA van der Pas MH de Lange-de Klerk ES. COLOR II Study Group. A randomized trial of laparoscopic versus open surgery for rectal cancer. N Engl J Med (2015) 372(14):1324–32. doi: 10.1056/NEJMoa1414882 25830422

[3] McDermott FT Hughes ES Pihl E Johnson WR Price AB. Local recurrence after potentially curative resection for rectal cancer in a series of 1008 patients. Br J Surg (1985) 72(1):34–7. doi: 10.1002/bjs.1800720115 3967128

[4] Hashiguchi Y Muro K Saito Y Ito Y Ajioka Y Hamaguchi T. Japanese Society for Cancer of the Colon and Rectum. Japanese Society for Cancer of the Colon and Rectum (JSCCR) guidelines 2019 for the treatment of colorectal cancer. Int J Clin Oncol (2020) 25(1):1–42. doi: 10.1007/s10147-019-01485-z 31203527 PMC6946738

[5] Fujita S Mizusawa J Kanemitsu Y Ito M Kinugasa Y Komori K. Mesorectal excision with or without lateral lymph node dissection for clinical stage II/III lower rectal cancer (JCOG0212): A multicenter, randomized controlled, noninferiority trial. Ann Surg (2017) 266(2):201–7. doi: 10.1097/SLA.0000000000002212 28288057

[6] Bosset JF Calais G Mineur L Maingon P Stojanovic-Rundic S Bensadoun RJ. Fluorouracil-based adjuvant chemotherapy after preoperative chemoradiotherapy in rectal cancer: long-term results of the EORTC 22921 randomised study. Lancet Oncol (2014) 15(2):184–90. doi: 10.1016/S1470-2045(13)70599-0 24440473

[7] Bosset JF Collette L Calais G Mineur L Maingon P Radosevic-Jelic L. Chemotherapy with preoperative radiotherapy in rectal cancer. N Engl J Med (2006) 355(11):1114–23. doi: 10.1056/NEJMoa060829 16971718

[8] Kim MJ Kim TH Kim DY Kim SY Baek JY Chang HJ. Can chemoradiation allow for omission of lateral pelvic node dissection for locally advanced rectal cancer? J Surg Oncol (2015) 111(4):459–64. doi: 10.1002/jso.23852 25559888

[9] Ogura A Konishi T Cunningham C Garcia-Aguilar J Iversen H Toda S. Neoadjuvant (Chemo)radiotherapy with total mesorectal excision only is not sufficient to prevent lateral local recurrence in enlarged nodes: results of the multicenter lateral node study of patients with low cT3/4 rectal cancer. J Clin Oncol (2019) 37(1):33–43. doi: 10.1200/JCO.18.00032 30403572 PMC6366816

[10] Kodeda K Johansson R Zar N Birgisson H Dahlberg M Skullman S. Time trends, improvements and national auditing of rectal cancer management over an 18-year period. Colorectal Dis (2015) 17(9):O168–79. doi: 10.1111/codi.13060 26155848

[11] Yang X Gu C Hu T Wei M Meng W Wang Z. Indications and oncological outcomes of selective dissection for clinically suspected lateral lymph node metastasis in patients with rectal cancer based on pretreatment imaging. Tech Coloproctol (2021) 25(4):425–37. doi: 10.1007/s10151-020-02386-4 33585985

[12] Kawai K Shiratori H Hata K Nozawa H Tanaka T Nishikawa T. Optimal size criteria for lateral lymph node dissection after neoadjuvant chemoradiotherapy for rectal cancer. Dis Colon Rectum (2021) 64(3):274–83. doi: 10.1097/DCR.0000000000001866 33395141

[13] Zhou S Jiang Y Pei W Zhou H Liang J Zhou Z. Neoadjuvant chemoradiotherapy followed by lateral pelvic lymph node dissection for rectal cancer patients: A retrospective study of its safety and indications. J Surg Oncol (2021) 124(3):354–60. doi: 10.1002/jso.26509 33882149

[14] Taylor FG Quirke P Heald RJ Moran BJ Blomqvist L Swift IR. Magnetic Resonance Imaging in Rectal Cancer European Equivalence Study Study Group. Preoperative magnetic resonance imaging assessment of circumferential resection margin predicts disease-free survival and local recurrence: 5-year follow-up results of the MERCURY study. J Clin Oncol (2014) 32(1):34–43. doi: 10.1200/JCO.2012.45.3258 24276776

[15] Smith NJ Barbachano Y Norman AR Swift RI Abulafi AM Brown G. Prognostic significance of magnetic resonance imaging-detected extramural vascular invasion in rectal cancer. Br J Surg (2008) 95(2):229–36. doi: 10.1002/bjs.5917 17932879

[16] Nicholls RJ Zinicola R Haboubi N. Extramural spread of rectal cancer and the AJCC Cancer Staging Manual 8th edition, 2017. Ann Oncol (2019) 30(8):1394–5. doi: 10.1093/annonc/mdz147 31046085

[17] Wang P Zhou S Zhou H Liang J Zhou Z. Evaluating predictive factors for determining the presence of lateral pelvic node metastasis in rectal cancer patients following neoadjuvant chemoradiotherapy. Colorectal Dis (2019) 21(7):791–6. doi: 10.1111/codi.14595 30801862

[18] Shiraishi T Sasaki T Tsukada Y Ikeda K Nishizawa Y Ito M. Radiologic factors and areas of local recurrence in locally advanced lower rectal cancer after lateral pelvic lymph node dissection. Dis Colon Rectum (2021) 64(12):1479–87. doi: 10.1097/DCR.0000000000001921 34657076

[19] Zhou S Jiang Y Pei W Liang J Zhou Z. Prognostic significance of lateral pelvic lymph node dissection for middle-low rectal cancer patients with lateral pelvic lymph node metastasis: a propensity score matching study. BMC Cancer (2022) 22(1):136. doi: 10.1186/s12885-022-09254-4 35109810 PMC8812196

[20] Taylor FG Quirke P Heald RJ Moran B Blomqvist L Swift I. One millimetre is the safe cut-off for magnetic resonance imaging prediction of surgical margin status in rectal cancer. Br J Surg (2011) 98(6):872–9. doi: 10.1002/bjs.7458 21480194

[21] PelvEx Collaborative. Factors affecting outcomes following pelvic exenteration for locally recurrent rectal cancer. Br J Surg (2018) 105(6):650–7. doi: 10.1002/bjs.10734 29529336

[22] Jayne DG Thorpe HC Copeland J Quirke P Brown JM Guillou PJ. Five-year follow-up of the Medical Research Council CLASICC trial of laparoscopically assisted versus open surgery for colorectal cancer. Br J Surg (2010) 97(11):1638–45. doi: 10.1002/bjs.7160 20629110

[23] Bhangu A Ali SM Brown G Nicholls RJ Tekkis P. Indications and outcome of pelvic exenteration for locally advanced primary and recurrent rectal cancer. Ann Surg (2014) 259(2):315–22. doi: 10.1097/SLA.0b013e31828a0d22 23478530

[24] Cercek A Roxburgh CSD Strombom P Smith JJ Temple LKF Nash GM. Adoption of total neoadjuvant therapy for locally advanced rectal cancer. JAMA Oncol (2018) 4(6):e180071. doi: 10.1001/jamaoncol.2018.0071 29566109 PMC5885165

[25] Fokas E Allgäuer M Polat B Klautke G Grabenbauer GG Fietkau R. Randomized phase II trial of chemoradiotherapy plus induction or consolidation chemotherapy as total neoadjuvant therapy for locally advanced rectal cancer: CAO/ARO/AIO-12. J Clin Oncol (2019) 37(34):3212–22. doi: 10.1200/JCO.19.00308 31150315

[26] Poulsen LØ Yilmaz MK Ljungmann K Jespersen N Wille-Jørgensen P. Local recurrence rate in a national Danish patient cohort after curative treatment for rectal cancer. Acta Oncol (2018) 57(12):1639–45. doi: 10.1080/0284186X.2018.1497299 30169998

[27] Shiraishi T Sasaki T Ikeda K Tsukada Y Nishizawa Y Ito M. Predicting prognosis according to preoperative chemotherapy response in patients with locally advanced lower rectal cancer. BMC Cancer (2019) 19(1):1222. doi: 10.1186/s12885-019-6424-4 31842797 PMC6916079

